# Real-Time PCR for Diagnosis of Oculoglandular Tularemia

**DOI:** 10.3201/eid1601.090793

**Published:** 2010-01

**Authors:** Max Maurin, Bernard Castan, Nathalie Roch, Brieuc Gestin, Isabelle Pelloux, Alexandra Mailles, Christophe Chiquet, Pascal Chavanet

**Affiliations:** Grenoble University Hospital, Grenoble, France (M. Maurin, B. Gestin, I. Pelloux, C. Chiquet); Joseph Fourier University, Grenoble (M. Maurin, B. Gestin, C. Chiquet); Auch Hospital, Auch, France (B. Castan); Dijon University Hospital, Dijon, France. (N. Roch, P. Chavanet); French Institute for Public Health Surveillance, Saint Maurice, France (A. Mailles)

**Keywords:** Francisella tularensis, tularemia, Parinaud oculoglandular syndrome, real-time PCR, diagnosis, France, bacteria, letter

**To the Editor:** Oculoglandular tularemia accounts for 3%–5% of all diagnosed tularemia cases (*1*). We report the diagnosis of this disease in 2 patients in France by real-time PCR.

Patient A, a 43-year-old woman, was referred in October 2006 to the infectious disease department of Auch Hospital (Auch, France). She had a fever (39°C) and severe conjunctivitis of the right eye that had evolved over 2 weeks despite administration of amoxicillin/clavulanate. The patient lived in a rural area endemic for tularemia and had regular contact with dogs and ring doves. She remembered harvesting mushrooms in a nearby forest a few days before onset of clinical symptoms. Physical examination showed a hyperemic and painful right conjunctiva, enlarged (0.5–1.5 cm in diameter) and tender preauricular and submandibular lymph nodes, and cellulitis of the right hemiface. Her condition rapidly improved after she received doxycycline and gentamicin.

Patient B, a 42-year-old woman, was referred in October 2008 to the infectious disease department of Dijon University Hospital (Dijon, France) for intermittent fever (38.5°C) and swollen left-sided pretragal and cervical lymph nodes, which had evolved for 3 weeks despite administration of amoxicillin, followed by pristinamycin and prednisone, and ciprofloxacin for 7 days. The patient remembered being scratched on the left hand by her dog several weeks earlier; the scratch healed spontaneously. She had recently walked in a nearby forest that was endemic for tularemia. Physical examination showed enlarged (2–3 cm in diameter), tender lymph nodes and bilateral conjunctivitis. Her condition improved after doxycycline therapy, but the pretragal lymph nodes were removed surgically in late November 2008 because of suppuration and necrosis. Ofloxacin was administered until January 2009 because of persistence of inflammation in cervical lymph nodes and suppuration with skin fistulization in the pretragal region.

Diagnostic investigations (Table) conducted at Grenoble University Hospital included serologic tests (microagglutination and indirect immunofluorescent antibody assay by using locally prepared *Francisella tularensis* subsp. *holarctica* antigen), culture, and 2 real-time PCRs. These PCRs were specific for insertion sequence *ISFtu2* or the Tul4 protein–encoding gene of *Francisella* sp. and used previously described primers, probes, an amplification protocol (*2*), and a LightCycler 2.0 apparatus (Roche, Meylan, France). We tested 5 μL of DNA extracted from clinical samples by using the QIAamp DNA Mini kit (QIAGEN, Hilden, Germany). Three negative controls (DNA-free water) and 1 positive control (DNA extracted from the *F*. *tularensis* subsp. *holarctica* LVS strain) were used for each PCR.

**Table:** Characteristics of the 2 patients in the study and test results for tularemia, France*

Characteristic	Patient A	Patient B
Age, y/sex	43/F	42/F
Blood leukocyte count at admission, cells/mm^3^	21,600	4,000–10,000
Blood granulocyte count at admission, cells/mm^3^	17,900	2,000–8,000
C-reactive protein level at admission, mg/L	51	39
Serologic test results for *Francisella tularensis*		
First serum sample, d	6	16
Microagglutination titer	<20	<20
Immunofluorescent IgM titer	<20	<20
Immunofluorescent IgG titer	<20	<20
Second serum sample, d	90	39
Microagglutination titer	80	160
Immunofluorescent IgM titer	160	320
Immunofluorescent IgG titer	160	320
Real-time PCR result for *ISFtu2* and *tul4*, sample (cycle threshold for each test, respectively)	Positive, conjunctival discharge (32.4 and 34.9)	Positive, pretragal lymph node (22.3 and 24.5)
*Francisella* sp. culture, sample	Positive, conjunctival discharge	Negative
*Francisella* subsp. identification	subsp. *holarctica*	subsp. *holarctica*

*Ig, immunoglobulin.

Seroconversion was found between acute-phase and convalescent-phase serum samples from both patients. A conjunctival cotton swab sample from patient A and pretragal lymph node suppuration and biopsy samples from patient B were positive for *F*. *tularensis* by both real-time PCRs. A *Francisella* sp. strain was isolated from the conjunctival discharge from patient A at Auch Hospital and Grenoble Hospital laboratories. Cultures were grown in a BioSafety Level 3 laboratory at Grenoble University Hospital because results of both PCRs were positive. Cultures of specimens from patient B were negative.

Both patients were infected with an *F. tularensis* subsp. *holarctica* strain. Infection was identified by PCR amplification and sequencing of the 16S rRNA gene (fD1 and rP2 primers) and the intergenic spacer region (FTitsFw 5′-ACCACGGAGTGATTCATGACTG-3′ and FTitsRv 5′-TCTCAATTGATTTCTCTTCCTAAGG-3′ primers) from the strain isolated from patient A and directly from the lymph node biopsy specimen from patient B.

Conjunctival inoculation of *F*. *tularensis* usually occurs by contact when a contaminated finger comes into contact with the eyes, e.g., after handling of an infected animal or tick (*3**,**4*), but the source of infection often remains undetermined, as for our 2 patients. Symptoms are not specific and correspond to Parinaud oculoglandular syndrome (*1*). Reported complications include keratitis, occasional corneal perforation, and lymph node suppuration; tonsillitis, cellulitis in nearby skin tissue, retinitis, erythema nodosum, and progression to systemic disease occur less frequently (*3**–**7*). A specific microbiologic diagnosis is needed for appropriate treatment because many microorganisms can cause Parinaud oculoglandular syndrome and clinical symptoms are not specific (*1**,**8*).

Fluoroquinolones are now considered first-line treatment for tularemia; β-lactam antimicrobial agents are not effective (*9*). Oculoglandular tularemia is a painful disease with a short incubation period (3–5 days), and results of serologic tests of acute-phase samples are often negative (*1**,**9*). Isolation of *F*. *tularensis* is difficult and hazardous to laboratory personnel (*1**,**9*). PCR-based techniques may enable a more rapid diagnosis (*1**,**9**,**10*). Heating clinical samples before testing prevents laboratory-acquired infections.

We report the use of real-time PCR for detection of *F*. *tularensis* from a conjunctival swab specimen. Many clinical laboratories are now equipped with this technology. Transport conditions of clinical samples (4°C, no transport medium, 24–48 h) are not restrictive. When compared with PCR, real-time PCR does not require post-PCR processing, enabling a faster turn-around time.

Oculoglandular tularemia is a rare but underestimated disease. Real-time PCR detection of *F*. *tularensis* DNA from conjunctival swab suspensions now provides a rapid, noninvasive, sensitive, and specific diagnosis of oculoglandular tularemia. This assay enables early establishment of specific antimicrobial drug therapy and poses no risk of infection for laboratory staff.
